# Supplementary material for: Total plaque score helps to determine follow-up strategy for carotid artery stenosis progression in head and neck cancer patients after radiation therapy
Source: PLoS One. 2021 Feb 12;16(2):e0246684. doi: 10.1371/journal.pone.0246684 (PMC7880459; doi:10.1371/journal.pone.0246684)
Supplement: Attachment — Submitted filename: letter to the reviewer_1224.docx [file pone.0246684.s002.docx]

Dear Paula Boaventura

Academic Editor, PLOS ONE

Thank you for your nice and detailed review. We’ve provided a point-by-point revision and our responses to all of your comments. The reasons and revisions are provided in the following contents. In the revised manuscript, all the changes are highlighted. We deeply appreciate your valuable review, which stimulated a more thorough consideration of the essay. Thank you very much.

Sincerely Yours,

Yeu-Jhy Chang, MD

Stroke Center and Department of Neurology

Chang Gung Memorial Hospital, Linkou Medical Center and College of Medicine, Chang Gung University, Taoyuan, Taiwan

No. 5, Fu-Hsing ST. Kueishan, Taoyuan, 33333 Taiwan

Tel: 886-3-3281200 ext 8340

Fax: 886-3-3288849

**Editor’s comment**

1. The manuscript will need extensive English editing.

Answer: Thank you, we’ve tried our best to improve the English editing, including the English revision by the native speaker.

2. Please ensure that your manuscript meets PLOS ONE's style requirements, including those for file naming.

Answer: Thank you, we’ve revised the manuscript according to PLOS ONE's style requirements.

3. Please provide **additional details** regarding participant consent. In the ethics statement in the Methods and online submission information, please ensure that you have specified what type you obtained (for instance, **written or verbal, and if verbal, how it was documented and witnessed**). If your study included minors, state whether you obtained consent from parents or guardians. If the need for consent was waived by the ethics committee, please include this information.

Once you have amended this/these statement(s) in the Methods section of the manuscript, please add the same text to the “Ethics Statement” field of the submission form (via “Edit Submission”).

Answer: Thank you for your reminding. We’ve added this in the manuscript.

**Materials and Methods,** page 4, line 12-14

The study was approved by the Ethics Institutional Review Board of Chang Gung Memorial Hospital (IRB No. 100-4153B). All of the included patients signed written informed consent forms.

**Reviewers' comments:**

Reviewer #1: The study was well-conducted with a reasonable analysis. The manuscript will likely merit publication with revisions.

1. The authors state that all data are fully available without restriction. Please publish the full dataset without identifying items to accompany the manuscript. If the datasets are too large to be supplementary files, then please publish in a separate repository, such as Zenodo or Mendeley.

Answer: Thank you for your comment. After discussing with the Ethics Institutional Review Board of out hospital, we’ve provided the data those could be accessible in the supplementary file.

2. Keywords

Since some search engine list only the title and keywords, choose terms that are not included in the title.

Answer: Thank you for your wonderful suggestion. We’ve added one keyword.

**Keywords:** Carotid artery stenosis, carotid duplex ultrasound, head and neck cancer, plaque scores, radiation therapy, radiation vasculopathy.

3. The authors note that HNC is common in Chinese populations. How many of their participants had Han ancestry? Does Han ancestry play a role in the outcomes if introduced to their models? Please identify the Han Chinese heritage of each participant to include in the multivariate logistic regression models.

Answer: Thank you for your expert opinion. In Taiwan, more than 95 percent of the population claiming Han ancestry (<https://www.taiwan.gov.tw/content_2.php>). In our study, all recruited patients were Han Chinese. It would be difficult to discuss the role of Han Chinese heritage in clinical outcomes.

We revised the manuscript accordingly.

**Results**, page 7, line 23-24

Between 2013 and 2014, we prospectively screened 361 Han Chinese patients with HNC who received RT at our hospital.

**Discussion**, page 12, line 12-14

We stated this in the limitation paragraph.

Fourth, the single-center nature of this study could have led to patient selection bias. Fifth, the generalizability of our conclusions to other ethnicities remains uncertain.

4. Figure 3. Legend Define "cum" ordinate.

Answer: Thank you for your suggestion. We’ve revised the ordinate of this figure. Besides, due to one figure was suggested to be added by another reviewer, figure 3 was renamed as figure 4.

New ordinate of this figure is “**Cumulative CAS progression-free rate”.**

**Figure 4.** Multivariate-adjusted survival curves estimating the time to CAS progression. The incidence rate of CAS progression was significantly higher in the HP group than in the LP group.

CAS, carotid artery stenosis; HP, high plaque score; LP, low plaque score; TPS, total plaque score.

Reviewer #2: This is a cogent, nicely composed, well rationalized, and potentially informative study intending to assess the predictive value of readily available ultrasonographic evaluation of patients with radiation induced carotid injury following HnN cancer. I would applaud the authors for **attempting to study a widely available, easily repeatable, and, generally speaking, affordable methodology for this purpose**. I do see very strong potential for its applications, but a few criticisms come to mind which I do believe are likely addressable. In all I believe the study would be of interest of the readership.

Answer: Thank you for your suggestion. We will keep working hard.

1. I do believe some ground truth comparison to cross-sectional angiographic methods that offer a more legitimate structural assessment would have been a complement. I do understand that this may not be possible in the entirety of the cohort, but even if a subset has such imaging, whether CTA, cath, or MRI , it would greatly contribute

Answer: Thank you for your suggestion. These are indeed important issues. However, due to the limitation of this study, we could only address this in the limitation paragraph. In the future study, we will endorse this suggestion into study protocol.

**Discussion,** page 11, line 26- page 12, line 9

First, CDU studies may not be a precise method to evaluate the degree of stenosis and plaque features. Confounding factors caused by the technique may have influenced the study results, and thus limited the interpretation of CAS progression. In addition, advanced cerebrovascular images including computerized tomography angiography, magnetic resonance angiography, or conventional angiography were not routinely arranged as a confirmatory study when CAS progression was detected by CDU. This may also have limited the interpretation of the study results. Second, baseline vascular status may have confounded the conclusions drawn from our results. A higher TPS or CAS before RT may have further worsened the clinical progression and may have led to bias in this study. However, in real-world practice, primary physicians may not know the vascular status of the carotid arteries prior to RT. Further cohort studies with more strict follow-up protocols and confirmatory vascular imaging studies are still needed to validate our suggested follow-up strategy.

2. I believe the longitudinal follow up is still Somewhat short term, and truly disambiguating in the arc of disease in different patients would require more regular and longer-term evaluation. I understand that this may be considered hypothesis building data for know and I hope the authors can emphasize that. Completely shifting practice paradigms based on what our somewhat preliminary results should be done only with immense circumspection

Answer: Thank you for your suggestion. These are quite important. We’ve added this into the limitation paragraph.

**Discussion,** page 12, line 14-16

Lastly, a longer follow-up duration may provide more conclusive answers. Completely shifting practice paradigms based on what our preliminary results should be done only with immense circumspection.

3.Lastly, imaging examples are completely lacking. Keep in mind that this is a highly operator dependent technique, and if anything I would suggest a large number of imaging examples including those that might highlight pitfalls or limitations.

Answer: Thank you for your suggestion. These are quite important. We’ve added one figure (figure 2) showing imaging examples.

**Results**, page 8, line 3-5

Of these 217 patients, 209 (96.3%) received CDU follow-up for more than 18 months after enrolment. We first recorded the presence and evaluated the predictors of CAS progression (Figure 2).

**Figure 2.** An illustrated case with carotid artery stenosis progression.

This figure shows typical carotid duplex ultrasound findings in a patient after radiation therapy. Prominent segmental changes were noted in bilateral common carotid arteries at the time of enrolment (A-C). Follow-up carotid duplex ultrasonography of the same patient 2 years later (D-F) demonstrated significant progression of stenosis (>50%) in the left common carotid artery (D) with hemodynamic changes (E).

Reviewer #3: The authors report the results of 217 such patients who have undergone carotid doppler ultrasound. The studies were reviewed by blinded trained radiologists. The inter-rater reliability was > 90% agreement.

1. (HOW WERE THE REMAINING DISAGREEMENTS HANDLED).

Answer: thank you for your comment.

The results of the CDU were read by single blind neurologist (CLC) when performing this retrospective analysis. Dr. CLC did not know the status of each enrolled patient. We compared Dr. CLC’s CDU results to the official reports of the CDU documented at the time of examination (between January 1, 2013 and December 31, 2014). There was no discordance between the official reports and Dr. CLC’s documentation. We stated this in the revised manuscript. Besides, “> 90%” was not the “inter-rater reliability” of CDU reporting. Instead, “> 90%” was the accuracy data comparing CDU results and conventional angiography when we set-up our CDU laboratory in 1992. We added the reference (Ref. 16) for this accuracy data.

**Materials and Methods,** page 5, line 19-23

The investigators who performed and read (CLC et al.) the baseline and follow-up CDU were blinded to the patients’ clinical conditions. Agreement was achieved in all CDU results between the investigators. Our CDU laboratory has an overall diagnostic accuracy rate of >90% for carotid stenosis [15, 16].

**Newly added reference:**

16.	Tseng KY, Lee TH, Ryu SJ, Chen ST. Correlation between sonographic and angiographic findings of extracranial carotid artery disease. Zhonghua yi xue za zhi = Chinese medical journal; Free China ed. 1992;50(4):302-6. Epub 1992/10/01. PubMed PMID: 1334789.

2. The authors report that high initial plaque score of >/= to 7 is very important in determining likelihood of progression in carotid stenosis following radiation therapy. (PLEASE REPORT THE TRANSDUCER ANGLE AT WHICH THE VELOCITIES WERE ACQUIRED FOR THE INTIIAL WORK UP AND FOR THE FOLLOW-UP SESSION. DIFFERENCES IN THE ANGLE COULD ARTIFICIALLY ELEVATE THE MEASURED ENDOVASCULAR FLOW VELOCITIES AND ERRONEOUSLY LEAD ONE TO INTERPRET INCREASING STENOSIS. PLEASE PROVIDE THIS INFORMATION.

Answer: Thank you for your reminding, we’ve revised the manuscript accordingly.

**Materials and Methods,** page 5, line 17-18

The degree of CAS was examined in the CDU study according to standard ultrasound criteria. The percentage of maximum stenosis in longitudinal views was determined using computer-assisted measurements of the 1-residual lumen diameter/vessel diameter × 100. The angle of insonation was set at 60 degrees during the flow velocity examination. Peak systolic velocities ≥120 cm/s were used to identify ≥50% CAS [14].

Thank you for the reviewers’ constructive and valuable advice and comments.

We hope the revised version could be more readable and can meet the requirements of PLOS ONE.

Best regards

Yeu-Jhy Chang, MD

Stroke Center and Department of Neurology

Chang Gung Memorial Hospital, Linkou Medical Center and College of Medicine, Chang Gung University, Taoyuan, Taiwan
