# Supplementary material for: Total plaque score helps to determine follow-up strategy for carotid artery stenosis progression in head and neck cancer patients after radiation therapy
Source: PLoS One. 2021 Feb 12;16(2):e0246684. doi: 10.1371/journal.pone.0246684 (PMC7880459; doi:10.1371/journal.pone.0246684)
Supplement: Attachment — Submitted filename: letter to the editor_minor revision.docx [file pone.0246684.s003.docx]

Paula Boaventura, PhD
Academic Editor
PLOS ONE

Thank you for your nice and detailed review. We’ve provided a point-by-point revision and our responses to all of your comments. The reasons and revisions are provided in the following contents. In the revised manuscript, all the changes are highlighted. We deeply appreciate your valuable review, which stimulated a more thorough consideration of the essay. Thank you very much.

Sincerely Yours,

Yeu-Jhy Chang, MD

Stroke Center and Department of Neurology

Chang Gung Memorial Hospital, Linkou Medical Center and College of Medicine, Chang Gung University, Taoyuan, Taiwan

No. 5, Fu-Hsing ST. Kueishan, Taoyuan, 33333 Taiwan

Tel: 886-3-3281200 ext 8340

Fax: 886-3-3288849

Reviewers' comments:
Reviewer #1: The authors assessed the utility of a total plaque score to predict carotid artery stenosis progression in patients with head and neck cancer after radiation.
The study was well-conducted with a reasonable analysis.
The manuscript will likely merit publication with revision to facilitate interpretation by clinicians.
Page 28 Figure 2 Legend
Please state the **age and sex** of the participant.

Answer: Thank you for your suggestion. We’ve added the age and sex of the illustrated case.

**Figure legends, figure 2**

This figure shows typical carotid duplex ultrasound findings in a 69-year-old male patient after radiation therapy.

Reviewer #2: Thank you for making the requested additions and clarifications. and its current form of this manuscript, while in park comprising preliminary or hypothesis building data is likely to offer some valuable insights regarding the intersection of credit disease and radiation – treated head and neck cancer patients. The largest areas for future clarification are cross reference with advanced structural cross-sectional angiographic imaging, the presence of longitudinal data to assess true progression over a longer period of time, and a better understanding of baseline variables in these patients.

Answer: Thank you for your kind comment, which indeed help us to design a better study to answer this question. We’ve revised the last paragraph of our discussion accordingly.

**Discussion, page 12, line 17-20**

Completely shifting practice paradigms based on what our preliminary results should be done only with immense circumspection. In the future, we may need a cross reference with advanced structural cross-sectional angiographic imaging, a longitudinal data to assess true progression over a longer period of time, and more comprehensive baseline variables in these patients for further clarification of our conclusions.

Reviewer #3: My concerns about potentially suboptimal transducer angles contributing to elevated endovascular velocities was satisfactorily addressed.

Answer: Thank you for your wonderful help. Your excellent comments indeed make our manuscript more readable.

Thank you for the reviewers’ constructive and valuable advice and comments.

We hope the revised version could be more readable and can meet the requirements of PLOS ONE.

Best regards

Yeu-Jhy Chang, MD

Stroke Center and Department of Neurology

Chang Gung Memorial Hospital, Linkou Medical Center and College of Medicine, Chang Gung University, Taoyuan, Taiwan
